# Effect of butorphanol-soaked nasal packing after endoscopic nasal surgery: a double-blind, randomized, placebo-controlled trial

**DOI:** 10.1016/j.bjorl.2023.101369

**Published:** 2023-11-22

**Authors:** Jiamei He, Qingyu Xiao, Yu Shuai, Xiaoli Liu, Shaohui Zhuang

**Affiliations:** a The First Affiliated Hospital of Shantou University Medical College, Department of Anesthesiology, Shantou City, Guangdong Province, China; b The Second Affiliated Hospital of Zunyi Medical University, Department of Anesthesiology, Zunyi, Guizhou Province, China

**Keywords:** Opioids, Nasal administration, Postoperative pain, Sleep quality

## Abstract

**Objective:**

This study was designed to investigate the effect of butorphanol-soaked nasal packing on analgesia and sleep quality in patients undergoing bilateral endoscopic nasal surgery.

**Methods:**

Sixty-six patients were enrolled and randomly allocated into three groups: group B1 (butorphanol 0.03 mg/kg), group B2 (butorphanol 0.04 mg/kg) and group N (control group). The primary outcome was postoperative pain scores evaluated by a Visual Analogue Scale (VAS) at 2 h (T1), 8 h (T2), 24 h (T3) and 48 h (T4) after surgery. Secondary outcome was postoperative sleep quality measured using Subjective Sleep Quality Value (SSQV).

**Results:**

Postoperative VAS scores of butorphanol groups were significantly lower than the control group at T2, T3 and T4. VAS scores at each time point did not differ between groups B1 and B2. On the first and second nights after surgery, SSQV was higher in butorphanol groups than in the control group. There were no significant differences in SSQV1 and SSQV2 between group B1 and group B2. The incidence of respiratory depression, dizziness, agitation and rescue analgesic use did not show difference among three groups.

**Conclusions:**

Butorphanol-soaked nasal packing can reduce pain and improve sleep quality after bilateral endoscopic nasal surgery without increasing adverse effects. A concentration of 0.03 mg/kg may be appropriate for clinical application.

**Level of Evidence:**

Level 1B.

## Highlights

• Butorphanol-soaked nasal packing can reduce pain and improve sleep quality.
• Butorphanol-soaked nasal packing does not increase adverse effects after surgery.
• 0.03 mg/kg butorphanol may be more appropriate for clinical use than 0.04 mg/kg.

## Introduction

Postoperative pain is an important symptom following nasal endoscopic surgery,^1^ which is related to both surgical trauma and nasal packing.^2^, ^3^ It has been shown that the use of nasal packing significantly increases pain experienced during the postoperative period.^4^, ^5^, ^6^

Postoperative pain may interfere with breathing, sleep quality, and patient satisfaction.^1^

Our previous study has proved that dexmedetomidine-soaked nasal packing not only offers effective analgesia but also improves postoperative sleep quality in patients undergoing bilateral endoscopic nasal surgery.^7^ Butorphanol tartrate, a mixed agonist and antagonist of opioid receptor, produces an analgesic effect by agonizing the κ receptor.^8^ Butorphanol has analgesic effects similar to morphine but significantly fewer adverse reactions such as respiratory inhibition.^9^ Besides analgesia, butorphanol exerts the effect of sedation through agonizing κ opiate receptors without tolerance.^10^ Therefore, we assumed that butorphanol-soaked nasal packing can reduce pain and improve sleep quality after bilateral endoscopic nasal surgery in adults.

## Methods

This study was approved by local ethics committee and registered at the Chinese Clinical Trial Registry (http://www.chictr.org; registration number: ChiCTR2000028880, date of trial registration: Jan 5th 2020). Informed consent was obtained from all patients prior to the study. The methodology of this study followed the World Medical Association’s Declaration of Helsinki.

### Patients

Patients aged 18–65 years who underwent elective bilateral endoscopic nasal surgery with a Body Mass Index (BMI) ranging from 18 to 30 kg/m^2^ and American Society of Anesthesiologists (ASA) grade I or II were eligible for recruitment. Exclusion criteria included asthma, respiratory infections within two weeks, obstructive sleep apnea syndrome, alcohol and opioid abuse, use of sedatives or analgesics, severe insomnia or mental disorders and illiteracy.

### Sample size estimation

The incidence of postoperative pain (visual analogue scale, VAS score > 3) within the first 24 h after endoscopic nasal surgery was 79%.^11^ We assumed that the incidence of postoperative pain (VAS score > 3) was 40% in group B1 (butorphanol 0.03 mg/kg) and 30% group B2 (butorphanol 0.04 mg/kg) with a two-sided significance level (α) of 0.05 and power (β) of 80%. Eighteen patients in each group (54 patients in total) were required to obtain a significant result. To compensate for any possible data loss, 66 cases were enrolled.

### Randomization

Patients were assigned to three groups (B1, B2 and N) using a computer-generated list of random numbers. Total dose of butorphanol did not exceed 4 mg, which was the maximum dose for a single intravenous injection.^12^ All solutions were colorless and configured with normal saline to a volume of 10 mL.

### Study design

General anesthesia was standardized for all participants with intravenous induction of propofol (2 mg/kg), sufentanil (0.4 μg/kg), and cisatracurium (0.2 mg/kg). Tracheal intubation was performed 2.5 min later. Anesthesia maintenance was achieved by propofol (4–8 mg/kg/h for a bispectral index/BIS range of 40–60) and remifentanil (0.15‒0.20 μg/kg/h). Cisatracurium (0.05 mg/kg) was administered intermittently for muscle relaxation. At the end of the surgery, propofol and remifentanil infusions were discontinued. Dezocine (5 mg) and tropisetron (4 mg) were administered intravenously. Patients were transferred to the Post-Anesthesia Care Unit (PACU) where they were monitored, extubated, and sent back to the wards.

All patients received two Polyvinyl Alcohol (PVA) sponges (Merocel, Medtronic Xomed, Jacksonville, FL, USA 32216) as nasal packings at the end of the surgical procedure. The preformulated solution was injected into the sponges averagely (5 mL at each side) by a surgeon blinded to the agent.

### Study measurements

Pain intensity was assessed using the Wong-Baker Scale combined with a visual analogue scale (VAS, a 10-point scale with 0 indicating no pain and 10 indicating the worst imaginable pain). Pain evaluations were performed at 4 time points: 2 h (T1), 8 h (T2), 24 h (T3) and 48 h (T4) after surgery.

Sleep quality on the first night after surgery and the second night after surgery was evaluated using the Subjective Sleep Quality Value (SSQV), a scale ranging from 0 to 100, with higher scores indicating better sleep quality.

In addition, preoperative Pittsburgh Sleep Quality Index (PSQI), Total Symptom Score (TSS), recovery time (duration from entry into PACU to tracheal extubation), vital signs right after tracheal extubation, adverse events including respiratory depression (pulse oximetry < 90%), dizziness, nausea, vomiting and agitation, and the incidence of rescue analgesic use were also recorded.

### Statistical analysis

SPSS V.25.0 was used for statistical analysis. All data were tested for normality using the Shapiro-Wilk and Kolmogorov-Smirnov tests. Quantitative data with a normal distribution were expressed as mean ± SD and analyzed with independent *t*-test, whereas those with a non-normal distribution were expressed as median (IQR) and analyzed with Kruskal-Wallis test. Qualitative data were analyzed using χ^2^ test or Fisher exact test. A *p*-value < 0.05 was considered statistically significant.

## Results

**Figure 1:**
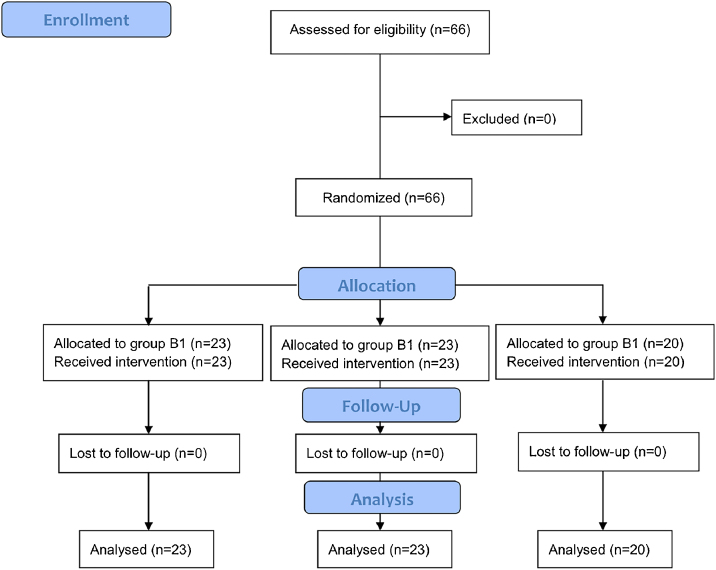
CONSORT flow chart representing enrolment, intervention allocation, follow-up, and data analysis.

Between April 2020 and February 2021, a total of sixty-six patients were enrolled in the study (Fig. 1). No significant difference was found among three groups in demographic data, baseline VAS, PSQI, TSS, types of surgery, operation time, volume of blood loss, anesthetics consumption or length of hospital stay (Table 1).

**Table 1:** Patient characteristics and basic surgical data.

	B1 (*n* = 23)	B2 (*n* = 23)	N (*n* = 20)	*p*
Age	35.74 ± 13.35	36.83 ± 13.85	41.00 ± 17.55	0.487
Male	17 (74%)	18 (78%)	15 (75%)	0.938
BMI (kg/cm^2^)	22.50 ± 2.65	22.99 ± 3.56	22.80 ± 2.99	0.973
Preoperative VAS, median (IQR)	0 (0, 0)	0 (0, 0)	0 (0, 0)	0.326
Preoperative PSQI, median (IQR)	1 (1, 2)	2(1, 2)	1(0, 2)	0.102
Preoperative TSS, median (IQR)	4 (2, 6)	4 (2, 6)	3 (1, 4)	0.105

Types of surgery				0.668
Correction of deviated nasal septum	28.57%	25.00%	37.14%	
Paranasal sinus opening	23.21%	19.64%	25.71%	
Resection of nasal polyps	16.07%	23.21%	11.43%	
External transfer of turbinate fracture	3.57%	5.36%	2.87%	
Reduction of nasal bone fracture	1.79%	0%	5.71%	
Partial turbinectomy	26.79%	26.79%	17.14%	

Operation time (min)	80 (65, 110)	75 (65, 105)	74 (46, 94)	0.350
Volume of blood loss (mL)	50 (20, 100)	50 (10, 200)	30 (13, 50)	0.537

Anesthetics consumption				
Propofol (mg)	680 (970, 510)	700 (600, 870)	700 (653, 973)	0.704
Remifentanil (μg)	1040 (900, 1300)	1100 (840, 1300)	965 (785, 1450)	0.809
Length of hospital stay (d)	7 (6, 8)	6 (6, 8)	7 (6, 8)	0.566

BMI, Body Mass Index; VAS, Visual Analogue Scale; PSQI, Pittsburgh Sleep Quality Index; TSS, Total Symptom Score.

**Figure 2:**
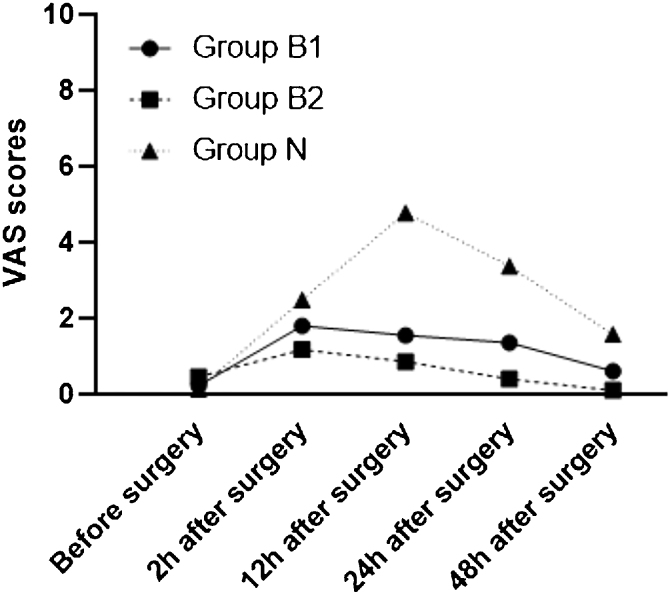
Postoperative VAS scores at different time points. VAS, Visual Analogue Scale.

**Figure 3:**
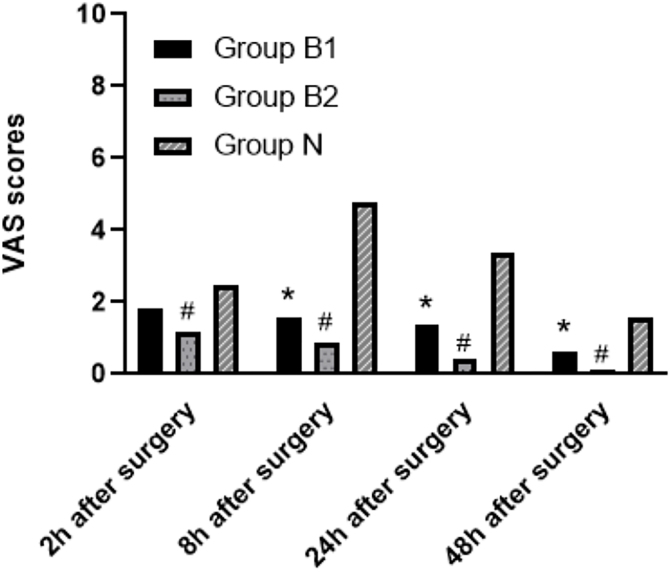
Comparison of VAS scores between groups. VAS, Visual Analogue Scale. * Mann-Whitney *U* test, *p* <  0.05 was considered statistically significant between group B1 and group N. # Mann-Whitney *U* test, *p* <  0.05 was considered statistically significant between group B2 and group N.

Fig. 2 presented VAS scores at T1, T2, T3 and T4. Postoperative VAS score of butorphanol groups were significantly lower than the control group at T2, T3 and T4. VAS score of group B2 at T1 was lower than that of group N. However, VAS scores at each time point were not different between group B1 and group B2 (Fig. 3).

All patients suffered from postoperative sleep disturbance after the operation. Table 2 revealed that sleep quality on the first night after surgery (SSQV1) was lower than that on the second night (SSQV2). On the first and second nights, SSQV was higher in butorphanol groups than in the control group. However, there were no significant differences in SSQV1 and SSQV2 between group B1 and B2.

**Table 2:** Postoperative subjective sleep quality values.

	B1	B2	N	*p*
SSQV1	60 (20, 80)	70 (30, 80)	20 (10, 38)	0.001
SSQV2	80 (50, 85)	85 (60, 90)	55 (23, 70)	0.004

No significant difference was found in recovery time and vital signs right after extubation among three groups. No respiratory depression occurred during the trial. The incidence of dizziness, agitation, and rescue analgesic use did not show difference among three groups. Incidence of nausea and vomiting was lower in group B1 than in group N (Table 3).

**Table 3:** Incidences of postoperative adverse effects.

	B1	B2	N	*p*
Respiratory depression	0 (0.00%)	0 (0.00%)	0 (0.00%)	1.000
Dizziness	12 (52.17%)	11 (47.83%)	7 (35.00%)	0.508
Nausea and vomiting	1 (4.34%)	3 (13.04%)	7 (35.00%)	0.023
Agitation	1 (4.34%)	3 (13.04%)	0 (0.00%)	0.116
Rescue analgesic use	3 (13.04%)	2 (8.70%)	5 (25.00%)	0.325

## Discussion

This study found that both 0.03 mg/kg and 0.04 mg/kg butorphanol-soaked nasal packing could decrease VAS score and increase SSQV without increasing recovery time or incidences of adverse events, which means that 0.03 mg/kg and 0.04 mg/kg butorphanol infiltrating nasal packing could effectively and safely reduce pain and improve sleep quality after endoscopic nasal surgery.

Current research on postoperative analgesia after endoscopic nasal surgery focuses on medications, such as opioids, nonsteroid anti-inflammatory drugs and α_2_ adrenergic agonists, or methods, including modification of nasal packing materials, punctuation and sphenopalatine nerve block. In recent years, nasal packing infiltration has been increasingly used for postoperative analgesia for endoscopic nasal surgery. Local anesthetics were most commonly applied for nasal packing. Studies of different teams showed that levobupivacaine, prilocaine or tetracaine infiltration before removal of nasal packs were safe methods to decrease discomfort and improve patient tolerability.^13^, ^14^, ^15^ Kim’s research also found that 50 μg of fentanyl-soaked packing significantly decreased postoperative pain with no observable adverse effects after closed nasal bone fracture reduction surgery.^16^ Study of our team has proved that dexmedetomidine-soaked nasal packing not only offers effective analgesia but also improves postoperative sleep quality in patients undergoing bilateral endoscopic nasal surgery.^7^ Butorphanol is a partial agonist of μ receptors and agonist of κ receptors, which demonstrates both analgesic and sedative effect. Adverse effect of somnolence brought by butorphanol might be a positive effect for sleep quality improvement after nasal surgery. Leander and Bailey found that intranasal administration of butorphanol had been shown to be a safe and effective alternative to intramuscular or intravenous administration because the nasal mucosa is highly vascularized, and the olfactory tissues provide a direct conduit to the central nervous system, bypass first-pass metabolism, and lead to an onset of action similar to IV drug administration.^17^, ^18^, ^19^ Result of this study confirmed that butorphanol-soaked nasal packing was also effective for postoperative analgesia of endoscopic nasal surgery.

We also explored the possible appropriate concentration of butorphanol for nasal packing. In this clinical trial, a large number of patients experienced moderate-to-severe pain in the first 24 h after surgery, which is consistent with the results of other studies on postoperative pain after nasal surgery.^20^, ^21^ And we found that VAS score of group B1 was lower than that of group N at 8 h, 24 h and 48 h postoperatively. However, for VAS score evaluated 2 h after the operation, there was no significant difference between the two groups. The possible reason was that butorphanol infiltration into nasal packing resulted in a relatively slow absorption. Therefore, for low concentration of butorphanol, the plasma concentration was not sufficiently high for pharmacological effects in the first 2 h. We also noticed that three groups of patients experienced only mild pain 2 h after surgery. This might be related to administration of dezocine, an opioid with an average half-life of 2.4 h. In addition, this study showed that the incidence of postoperative pain (VAS score > 3) within 24 or 48 h was similar between group B1 (21.7%) and group B2 (21.7%), which was much lower than that in group N (85%, *p* =  0.000). Therefore, for postoperative analgesia within 48 h, 0.03 mg/kg butorphanol for nasal packing may be adequate.

Postoperative pain can cause disturbance of circadian rhythm. Bilateral nasal obstruction with tight packing and altered breathing with mouth opening result in poor sleep quality, which could further lower the threshold of pain. Postoperative pain and sleep disorder are reciprocal. In this study, we also investigated the effect of butorphanol nasal packing on sleep quality. The result showed that All patients experienced sleep disorders after surgery. Sleep quality on the first night was worse than that on the second night. Butorphanol groups had higher scores of both SSQV1 and SSQV2. Additionally, the number of patients with poor sleep quality (SSQV < 30) in the control group was higher than that in butorphanol groups (group N, 75%; group B1, 35%; group B2, 30%). The number of patients with good sleep quality (SSQV > 90) was higher in butorphanol groups than in the control group (group N, 10%; group B1, 22%; group B2, 44%). However, there was no significant difference between group B1 and B2. Therefore, with respect to improvements in sleep quality, 0.03 mg/kg butorphanol for nasal packing is effective.

This study also showed that the incidence of postoperative nausea and vomiting was lower in group B1 (0.03 mg/kg) than group N. This result was similar to Yang’s research,^22^ where intranasal administration of 2 mg butorphanol in patients undergoing H-uvulopalatopharyngoplasty significantly reduced the occurrence of nausea and vomiting compared with the control group. Activation of μ receptors by opioid is associated with adverse effects of central nervous system, such as nausea and vomiting. As a partial agonist of μ receptors, butorphanol could possibly antagonize these undesired effects. However, we found that the incidence of postoperative nausea and vomiting in high concentration group (0.04 mg/kg) was not significantly different from the control group. A systematic review of randomized controlled trials also concluded that epidural, but not intravenous butorphanol reduced Postoperative Nausea and Vomiting (PONV).^23^ So low plasma concentration of butorphanol may play a positive role in reduction of PONV, Higher concentration may be related to more obvious adverse effects. In this study, 0.03 mg/kg was more effective in prevention of PONV than 0.04 mg/kg. Further investigation is required to confirm the underlying pharmacological mechanisms and the optimal concentration for reduction of PONV.

Butorphanol-soaked nasal packing may also be an alternative for postoperative pain management in patients with obesity. First, butorphanol is a partial agonist of μ receptors and agonist of κ receptors without dose-related respiratory depression,^1^ which may be an appropriate option for obese patients with a high risk of airway obstruction and hypoxia. Second, butorphanol-soaked nasal packing improved sleep quality probably because of both pain relief and mild sedation. Thus, extra hypnotics can be reduced or even avoided postoperatively, and the incidence of airway obstruction and hypoxia may decrease consequently. Third, this study proved that local administration (nasal packing) of even a low dose of analgesics was effective for pain control after nasal surgery, which reduced respiratory adverse events from traditional intravenous opioids. Furthermore, a recent study even found that butorphanol administration protected pulmonary function by improving oxygenation and reducing dead space ventilation in patients with obesity.^24^ Apart from the respiratory problems, obesity may also be associated with other clinical issues, such as cardiovascular diseases^25^, ^26^ and psychiatric disorders,^27^, ^28^ which could deteriorate due to pain and insomnia. Therefore, butorphanol-soaked nasal packing with satisfactory effects on pain control and sleep improvement may be clinically beneficial in obese patients.

This study proves that butorphanol-soaked nasal packing can effectively reduce pain and improve sleep quality after bilateral endoscopic nasal surgery without increasing adverse effects, which is a noninvasive and practical method of pain relief and sleep improvement for these patients. Comparison of two concentrations of butorphanol infiltrating nasal packing provides a possibly appropriate concentration of 0.03 mg/kg for clinical use. This double-blind, randomized controlled study was performed according to strict scientific protocols with reliable results and conclusions, which could be applied in clinical practice and bring actual benefits to patients.

However, this study has some limitations. First, we found that a low concentration (0.03 mg/kg) of butorphanol reduced pain and improved sleep quality after endoscopic nasal surgery, with less nausea and vomiting compared with high concentration (0.04 mg/kg). But whether even lower concentration can provide equally effective postoperative analgesia requires further investigation. Second, the study included only young adults with relatively good health conditions (ASA grade I or II). Whether children, elderly patients or patients with severe underlying diseases who undergo endoscopic nasal surgery would benefit from this nasal packing remains unclear, which is what we will continue to explore in the future. Last but not least, Polyvinyl Alcohol (PVA) sponges were used for nasal packing. Further research could focus on other materials like Nasopore, vaseline gauze or polyurethane foam as infiltration carriers for postoperative pain control.

## Conclusion

Butorphanol-soaked nasal packing can reduce pain and improve sleep quality after bilateral endoscopic nasal surgery without increasing adverse effects. A concentration of 0.03 mg/kg may be appropriate for clinical application.

## Data sharing statement

All data generated or analyzed during this study were included in the published article. Further inquiries about the datasets can be directed to the corresponding author: Professor Shaohui Zhuang on reasonable request.

## Funding

This research did not receive any specific grant from funding agencies in the public, commercial, or not-for-profit sectors.

## Conflicts of interest

The authors declare no conflicts of interest.
